# Pathological Gambling and Alcohol Use Disorder

**Published:** 2002

**Authors:** Jon E. Grant, Matt G. Kushner, Suck Won Kim

**Affiliations:** Jon E. Grant, M.D., is a psychiatry resident, Matt G. Kushner, Ph.D. is an associate professor, and Suck Won Kim, M.D., is an associate professor, all in the Department of Psychiatry, University of Minnesota Medical School, Minneapolis, Minnesota

**Keywords:** pathological gambling, AODD (alcohol and other drug dependence), comorbidity, etiology, diagnostic criteria, disinhibition, impulsive behavior, ventral tegmental area, encephalopathy, naltrexone, genetic linkage, causal path analysis, treatment outcome

## Abstract

Problematic gambling is more common among people with alcohol use disorders (AUDs) (i.e., either alcohol abuse or dependence) compared with those without AUDs. This association holds true for people in the general population and is even more pronounced among people receiving treatment. No broadly accepted explanation for the link between problematic gambling and AUD currently exists. The available literature suggests that common factors may increase the risk for both conditions. For example, a defect of functioning in a particular brain system may underlie both conditions. This hypothesis should be further developed using brain imaging and psychopharmacological studies. Effective treatment and prevention will require additional research into relevant associations on both the event level (e.g., the effects of drinking on gambling behavior and vice versa) and the syndrome level (e.g., the relative onset and course of each condition among those who have either one or both disorders). A prudent interpretation of the available data suggests careful screening and treatment when necessary for problematic gambling among people with alcohol abuse and for alcohol abuse among people with gambling problems.

Pathological gambling (PG) is characterized by a persistent maladaptive pattern of gambling behavior. PG was first formally included in the *Diagnostic and Statistical Manual of Mental Disorders, Third Edition* (DSM–III) in 1980 (American Psychiatric Association [APA] 1980). Currently, PG is classified under the category “disorders of impulse control not elsewhere classified” (APA Committee on Nomenclature and Statistics 2000).

This article explores the association between pathological gambling and alcohol use disorders (AUDs) (i.e., the general name for either alcohol abuse or alcohol dependence). It first examines the separate and overlapping prevalences of PG and AUD as estimated by epidemiological surveys conducted in both community and clinical samples. The article then reviews the processes and mechanisms that might account for the frequent co-occurrence of these disorders. Finally, it examines what the co-occurrence of these disorders implies for treatment and highlights promising areas for future research.

Many terms have been used to describe people with problematic gambling behavior (see Cunningham-Williams and Cottler 2001). In this article, PG refers to pathological gambling as diagnosed using DSM diagnostic criteria. The term “disordered gambling behavior” is used to refer to problematic gambling behavior that is not defined by DSM diagnostic criteria. Note, however, that this term is not used as a means of identifying a less serious gambling problem compared with PG. Rather, this term is used to distinguish between problem gambling formally shown to meet the DSM criteria (PG) and all other cases of problematic gambling behavior (disordered gambling behavior).

## Epidemiology

### Pathological Gambling

During the 1990s, changes in State and local legislation encouraged the expansion of all types of wagering (e.g., casino gambling, lotteries, Internet gambling). As an apparent consequence, gambling and gambling-related problems are on the rise in the United States and Canada. A recent meta-analysis of 120 published studies estimated that 1.6 percent of adults in the United States and Canada meet the DSM criteria for pathological gambling at some point in their lives (Shaffer et al. 1999). Among people younger than 18 years of age, the current prevalence of PG is estimated to be 3.9 percent, with past-year rates for adults and adolescents estimated at 1.1 percent and 5.8 percent, respectively (Shaffer and Hall 1996).

Slightly lower prevalence rates of PG were found by a national study conducted by the National Opinion Research Center (NORC). This study found the lifetime prevalence of adult pathological gambling to be 0.9 percent, with past-year rates of 0.6 percent (NORC 1999).

Although studies using historical review (i.e., retrospective methodologies) have tended to point to a young age of onset of PG (Cunningham-Williams and Cottler 2001), more definitive studies that follow participants over time (i.e., prospective studies) to determine the natural course of PG have yet to be conducted. Studies looking at cross sections of different age groups have shown that PG prevalence rates appear higher for adolescents and young adults than for middle-aged and older adults (Shaffer and Hall 1996). Long-term studies are necessary, however, to clarify these findings.

### AUD

Based on DSM–III criteria, the Epidemiologic Catchment Area (ECA) survey estimated that 13.8 percent of adults in the United States meet the criteria for AUD at some time during their lives (Robins et al. 1991). According to findings from the National Comorbidity Survey (NCS) (Kessler et al. 1994), the estimated lifetime prevalence is 14.2 percent for alcohol dependence and 9.4 percent for alcohol abuse, based on criteria in the DSM–III–R (APA 1987). The National Longitudinal Alcohol Epidemiologic Survey (NLAES) found that over the 12 months preceding the survey, 4.4 percent of adults age 18 or older met the criteria for alcohol dependence and 3.0 percent met the criteria for alcohol abuse (Grant et al. 1994). In sum, between 7 percent and 14 percent of adults in the United States experience an AUD at some point in their lives.

The NCS found that the median age of onset was 19 years for alcohol abuse and 18 years for alcohol dependence (Kessler et al. 1997). The NLAES found that the risk for developing an AUD was substantially increased among those who started drinking before age 17 (24.5 percent lifetime prevalence) compared with those who started drinking at age 21 or 22 (10 percent lifetime prevalence) or at age 25 years or older (less than 4 percent lifetime prevalence) (Grant and Dawson 1997). In fact, the prevalence of alcohol abuse detected in that study declined with each increasing year of age of onset of drinking.

### Comorbid AUD and PG

Given the relative frequency of both PG and AUD, these conditions would be expected to co-occur in some cases by chance alone. However, there is evidence that disordered gambling behavior and AUD co-occur in U.S. and Canadian residents at a rate exceeding that expected by chance. As described below, studies of treatment populations found an increased risk of PG in alcoholism treatment patients and an increased risk for AUD in PG treatment patients.

#### Community Populations

A large epidemiological survey in Canada estimated that the relative risk for AUD is 3.8 times higher when disordered gambling behavior is present (Bland et al. 1993). A study conducted in the United States found that 44 percent of those with disordered gambling behavior also report a lifetime history of AUD (Cunningham-Williams et al. 1998), a rate that greatly exceeds general prevalence estimates for AUD (see above). In fact, these studies reported that as gambling severity increased, so did the risk for AUD, even when sociodemographic variables were controlled. Similarly, Feigelman and colleagues (1998) found that 26 percent of U.S. community-based respondents with disordered gambling behavior reported having experienced AUD or some other substance use disorder at some point during their lifetime.

These survey findings, considered against findings from the ECA survey showing that approximately 14 percent of U.S. adults experience AUD (above), indicate that, in the general population, the risk for AUD is two to four times higher for people with PG compared with those without PG. However, a more recent study found a substantially greater association between AUD and PG (Welte et al. 2001). This study reported an odds ratio for current alcohol dependence with current PG of 23.1 (Welte et al. 2001). That is, the odds of having a current alcohol dependence diagnosis were 23 times greater among those in the survey who had a PG diagnosis than for those with no PG diagnosis. It should be noted that this study focused on having a current (vs. lifetime) diagnosis and on alcohol dependence, rather than abuse. Similar to the study reported by Cunningham-Williams and colleagues (1998), Welte and colleagues (2001) also found that the prevalence of current PG increased with increasing alcohol consumption. Among those people with higher socioeconomic status, alcohol dependence and PG were even more strongly correlated (Welte et al. 2001).

#### Treatment Populations

Consistent with observations pertaining to comorbidities involving a variety of disorders (e.g., Kushner et al. 1990), comorbid PG and AUD tend to be more prevalent in treatment (vs. community) samples. One early study suggested that 17 percent of alcoholism treatment patients reported disordered gambling behavior (Haberman 1969). A later study of 100 inpatients with alcohol dependence found that 14 percent met the criteria for PG, and that an additional 14 percent suffered from subclinical gambling problems (Lesieur and Heineman 1988). In a study of 79 patients with alcohol dependence, 7 (8.9 percent) met the criteria for PG (Lejoyeux et al. 1999). Other researchers have estimated that 20 percent of patients undergoing substance abuse treatment have problems with gambling (Lesieur et al. 1986; Giacopassi et al. 1998). In a study of 276 patients consecutively admitted to a Veterans’ Administration Hospital for substance abuse, 33 percent met the DSM–III–R criteria for comorbid substance abuse and PG (Daghestani et al. 1996). Given that the lifetime prevalence of PG in the general population has been estimated to be anywhere from 1 percent to 6 percent (see above), these findings indicate a dramatic increase in risk for PG among alcoholism treatment patients.

Elevated rates of alcohol dependence in people receiving treatment for PG further support the importance of the association between PG and AUD. A recent study found that 23.2 percent of PG patients suffered from current AUD, and that 34.8 percent reported lifetime AUD (Ibanez et al. 2001). Similarly, a relatively large study of PG patients found that 27 percent suffered from lifetime AUD (Grant and Kim 2001). Earlier studies have reported that anywhere from 19 percent to 48 percent of PG patients have a lifetime or current alcohol problem (Roy et al. 1988; Linden et al. 1986; McCormick et al. 1984; Ramirez et al. 1984). Contrasted with risk estimates reviewed above, these findings support an approximate doubling to quadrupling of risk for AUD among PG treatment patients relative to the risk for people in the general community with no PG.

#### Moderating Variables

The data reviewed above lack detail concerning the potential influence of various sociodemographic variables on risk for comorbidity. Surveys estimate that approximately two-thirds of pathological gamblers are men (Volberg 1994). It is also well known that men are substantially more likely than women to develop an AUD (e.g., Helzer et al. 1991). Because men are more likely than women to experience both AUD and PG, the co-occurrence of these disorders by chance alone should be more common among men. In one of the few studies to examine gender differences in comorbidity rates, men with disordered gambling behavior were more likely to report drinking problems than were women with disordered gambling behavior (20 percent vs. 15 percent); however, this difference was not deemed statistically significant (Potenza et al. 2001).

Additional comorbidities involving other psychiatric disorders may further complicate understanding of the relationship between AUD and PG. For example, recent studies have shown that people with attention deficit hyperactivity disorder (ADHD) are at increased risk for developing a substance use disorder (Mannuzza et al. 1993; Biederman et al.1995). In addition, studies show that pathological gamblers have an increased prevalence rate of comorbid ADHD (Carlton et al. 1987; Specker et al. 1996). Although little relevant research has been conducted, a reasonable hypothesis for further study is that some psychiatric conditions may mark those who are more likely to develop both AUD and PG. Such a finding might also point toward factors (e.g., impulsivity associated with ADHD) common to the etiology of both AUD and PG.

Studies have also examined whether rates of PG differ among racial or ethnic groups (Cunningham-Williams and Cottler 2001). For example, research has shown higher rates of PG among Native Americans in alcoholism treatment compared with Caucasians (5.9 to 22 percent versus 0.8 to 7.3 percent) (Volberg and Abbott 1997; Elia and Jacobs 1993). The St. Louis site for the ECA study found a higher proportion of African-Americans represented among problem gamblers (31 percent) than among nonproblem gamblers (15 percent) (Cunningham-Williams et al. 1998). Importantly, however, a variety of characteristics (e.g., socioeconomic status) are correlated with race and ethnicity. That is, it is hard to interpret findings comparing racial/ethnic groups on comorbidity without also considering a wide range of variables that often correlate strongly with race/ethnic designations (e.g., social, economic, cultural, and geographic variables). Studies showing different base rates of AUD and PG across racial/ethnic groups suggest the potential importance of further study in this area.

As reported above, AUD and PG co-occur at a rate significantly exceeding that expected by chance in both general community and treatment samples. No explanation or cause for the association between AUD and PG is apparent or implied in the epidemiological studies reviewed thus far. The next section addresses this question.

## Temporal and Causal Relationships

When attempting to understand comorbidity, establishing the temporal relationship of the two disorders is intuitively appealing (i.e., which disorder started first?). However, clearly establishing and interpreting such relationships as a means of better understanding the nature of comorbid associations has proven challenging (e.g., Kushner et al. 2000). One disorder consistently preceding the other would be consistent with, but not proof of, a direct causal relationship. Heterogeneity in the order of onset, on the other hand, would be consistent with (but again, not proof of) the existence of a third variable (e.g., shared genetic factors or pathophysiology) serving as a common cause for both conditions. Further complicating the picture, causal associations may manifest on an event level (e.g., alcohol use may disinhibit a wide range of inappropriate behaviors including problematic gambling [e.g., Smart and Ferris 1996]) or on a syndrome level (e.g., a new onset of PG occurring, ostensibly as a substitute for drinking, following alcoholism treatment [e.g., Ingram-Smith 1967; Lesieur and Heineman 1988]).

In one of the few studies to examine the temporal pattern of disorder onset in comorbid PG and AUD, Cunningham-Williams and Cottler (2001) reported that PG began after comorbid nicotine, alcohol, and cannabis dependence in 56 percent to 68 percent of the cases. However, confidence in this finding is limited by the absence of similar studies against which to evaluate its reliability. Further, because information was collected in this study by asking participants to recall significant historical events (i.e., a retrospective design), confidence in its findings is further qualified. The ideal study design to assess patterns of onset for comorbid disorders would entail assessing people with neither, one, or both of the comorbid disorders at numerous time points (i.e., a prospective design). Ideally, such a study would follow participants through the ages of greatest risk for the onset of these disorders, and the assessment periods would be spaced closely enough to capture changes in relevant behaviors in near real time.

Until such studies are conducted in this area, researchers cannot confidently identify a typical pattern of comorbid disorder onset. However, based on the one relevant study identified (Cunningham-Williams and Cottler 2001), it would appear that neither PG nor AUD routinely precedes the other in cases of comorbidity. As noted, this pattern is most consistent with a common cause for both conditions. Potential common causes are described below.

## Processes and Features Common to PG and AUD

### Common Diagnostic Criteria

Addictive behaviors are broadly characterized by a number of features. These general characteristics include an intense desire to satisfy a need, a loss of control over the substance or behavior, compulsive thoughts about the substance or behavior, and engaging in the behavior despite negative consequences (World Health Organization [WHO] 1993). In fact, the DSM–IV defines the essential features of substance dependence as being “a cluster of cognitive, behavioral, and physiological symptoms, indicating that the individual continues to use the substance despite significant substance-related problems” (APA 1994, p. 176).

More so than other impulse control disorders, the criteria for PG share striking similarities with those for substance dependence (Lesieur and Rosenthal 1991). Reflecting the fact that the DSM–IV criteria include the concepts of preoccupation, loss of control, tolerance, and withdrawal, PG has been described as an “addiction without the drug” (Potenza et al. 2001). People with PG, like many people with AUD, experience an intense wish to engage in the behavior (Castellani and Rugle 1995). In fact, those with PG may even undergo withdrawal symptoms such as irritability and agitation (Wray et al. 1981) and experience the equivalent of tolerance (i.e., the need to gamble with larger amounts of money to attain the same “high”) (Wray et al. 1981; Blanco et al. 2001). Additionally, as can be the case for addictions involving a substance, pathological gamblers’ preoccupation with gambling can lead to the abandonment of other interests and negative social and occupational consequences (Lesieur 1979; Wray and Dickerson 1986). Although defining nondrug use behaviors such as PG as an addiction is not without controversy, a recent critical review of this topic concluded that mounting evidence supports such a conceptualization (Holden 2001).

Because the fact that they are addictive behaviors is fundamental to both AUD and PG, physical and psychological processes that drive addictive behaviors are likely candidates in the search for a common cause of these comorbid disorders (e.g., Holden 2001). The next section considers such processes.

### Common Neurobiological Processes Underlying Urges and Rewards

The repetitive use of alcohol or engagement in gambling following an urge may reflect a unitary process. That is, both behaviors may stem from the same underlying mechanism. Preclinical and clinical studies suggest that an underlying biological mechanism for urge-based disorders involves the processing of incoming reward inputs by a specific brain system. This system is the ventral tegmental area (VTA)/nucleus accumbens/orbital frontal cortex circuit. The VTA is a brain region containing cells (neurons) that release the brain chemical (neurotransmitter) dopamine, with target molecules (receptors) in the brain areas known as the nucleus accumbens and the orbital frontal cortex (Koob and Bloom 1988; Mogenson et al. 1980; Berridge 1996; Hyman 1993). This circuit is thought to influence behavior by modulating motivation that, at the level of subjective experience, is perceived as urges or cravings. Dopamine may also play a major role in the regulation of this region’s functioning (Kuhar et al. 1991; Self et al. 1996). In fact, researchers have theorized that dysregulation in the systems supporting the activities of dopamine and the neurotransmitter serotonin may be central in both AUD and PG (Comings et al. 1996; Blum et al. 1995). Further, evidence suggests that specific genetic variations in the gene for the dopamine D2 receptor (a specific binding molecule with which dopamine interacts) and the serotonin transporter gene may mediate, to some extent, individual differences in reward motivation and responsiveness (Potenza 2001; Ibanez et al. 2001). Therefore, common etiologic factors underlying AUD and PG may be partially genetic and mediated through nervous system functioning.

### Genetic and Environmental Factors

Researchers can estimate the extent of genetic versus environmental contributions to specific behaviors and conditions by contrasting their concordance between identical (i.e., monozygotic) and fraternal (i.e., dizygotic) twin pairs. In the only twin study that specifically examined these associations for PG and AUD, Slutske and colleagues (2000) reported that in a large male twin sample, 12 to 20 percent of the genetic variation in risk for PG and 3 percent to 8 percent of the nonshared environmental variation in the risk for PG was accounted for by risk for AUD.

Additionally, 64 percent of the co-occurrence between PG and AUD appears to be attributable to genes that simultaneously influence both disorders. Similarly, family studies have also found that study participants with PG tended to have first-degree relatives with AUDs (McElroy et al. 1992; Slutske et al. 2000). Although these findings have not been replicated, they suggest some overlap in the genetically transmitted underpinnings of both of these conditions. Notably, however, these findings also point to significant genetic and environmental risks for PG and AUD that are unique to each disorder.

### Responsiveness to Treatment

Based on the view that PG and AUD share common causal/maintaining factors, one could predict that these disorders would respond positively to the same treatments. In fact, various nonmedical treatment modalities that are effective in treating AUD are also useful in treating PG (e.g., 12-step approaches and cognitive behavioral therapies; Petry and Roll 2001). In addition, several influential psychosocial interventions for both conditions rely on a relapse prevention model. This model encourages abstinence by identifying patterns of abuse, avoiding or coping with high-risk situations, and making lifestyle changes that reinforce activities not related to substance use.

Researchers have only recently started to explore pharmacologic treatment approaches for PG. Several studies have shown promising results for the efficacy of selective serotonin reuptake inhibitors (SSRIs) (medications that affect the production and/or absorption of serotonin) in the treatment of PG (Hollander et al. 2000; Kim et al. in press; Zimmerman and Breen 2000; Blanco-Jerez 1999; de la Gandara 1999). The use of SSRIs in the treatment of AUD, however, has been less impressive (Kranzler et al. 1995; Kabel and Petty 1996; Angelone et al. 1998; Cornelius et al. 1997).

Naltrexone, which blocks the action of opioids (i.e., it is an opioid antagonist), has been effective in reducing the frequency and amount of drinking in patients with AUD (Volpicelli et al. 1992; O’Malley et al. 1992; Anton et al. 1999). Studies evaluating the efficacy of naltrexone in the treatment of PG have also demonstrated its benefit in reducing gambling urges (Crockford and el-Guebaly 1998; Kim et al. in press; Kim and Grant 2001). One pharmacological action of naltrexone is to inhibit the release of dopamine in the nucleus accumbens by restoring the neurotransmitter gamma-aminobutyric acid’s (GABA’s) inhibition of dopamine cells in the VTA (Broekkamp and Phillips 1979; Matthews and German 1984; Spanagel et al. 1992). As mentioned above, it has been postulated that symptoms of AUD and PG are modulated, in part, by the neural systems (that regulate pleasure) affected by naltrexone. Thus, the positive naltrexone treatment outcome found in AUD and PG provides evidence that systems affected by this drug play roles in both conditions.

## Alcohol Use Disinhibits Gambling Behavior

As described above, a number of processes might serve as common underlying mechanisms for PG and AUD, thereby promoting comorbidity. An alternative explanation for the frequent association of these disorders is that repetitive or problematic alcohol use might, itself, serve to increase the risk for PG. In fact, engaging in gambling while drinking is common (Lesieur et al. 1986). Further, evidence suggests that alcohol use can adversely affect cognitive processes, leading to poor judgment and increased risk-taking. For example, studies have shown that alcohol intake is associated with impaired decisionmaking (Baron and Dickerson 1999) and reduced self-reflection (e.g., considering the consequences of behavior) associated with risk-related judgments (Breslin et al. 1999). Alcohol might also increase risk-taking by restricting attention to only the most salient and immediate cues (Steele and Josephs 1988), leading to less regard for the actual odds of a gamble and past gambling losses.

In spite of the prima facia evidence for an association between drinking and gambling behavior, studies to date are mixed in their support of the hypothesis that alcohol frequently causes problematic behavior leading to PG. Consistent with the hypothesis, one study found that alcohol intake was associated with greater spending on gambling activities and with gambling problems (Smart and Ferris 1996). Another study found a significant increase in the willingness to gamble when alcohol was consumed, but only when the amount consumed was limited to about two drinks. The effect disappeared at higher doses (Breslin et al. 1999). Several studies do not support the notion that alcohol use affects willingness to gamble (Cutter et al. 1973; Meier et al. 1996). One study also showed that risk-taking while gambling is not increased by alcohol consumption (Breslin et al. 1999). These data appear to call into question the intuitively appealing hypothesis that problem gambling follows from problem drinking when intoxication promotes excessive or highly risky gambling behavior. Further study in this area is necessary.

## Gambling Promotes Alcohol Use

Another possible association between pathological gambling and alcohol use disorders is that PG may promote AUDs. For example, if people are more likely to drink while gambling, then it might follow that the risk for alcohol problems increases when frequent gamblers are regularly exposed to alcohol. Very little empirical work has addressed this question. One exception is a recent study by Stewart and colleagues (in press). They found that frequent gamblers self-administered more alcohol in a simulated gambling situation than did matched study participants engaged in a control activity. These findings are provocative and invite more research to determine the extent to which gambling behavior promotes alcohol consumption.

## Treatment Implications

Regardless of the specific causal association linking PG and AUD, the fact that they frequently co-occur raises important treatment issues. Treatment of either AUD or PG could be complicated or even compromised by the presence of the other untreated condition (e.g., Kranzler and Liebowitz 1988). Treating one disorder alone may not be effective if the second disorder is exerting a causal or maintaining influence on the treated condition. Even in the more likely event that AUD and PG are associated via the influence of a third variable that can promote both disorders, treating one but not the other condition would be potentially problematic. For example, the circumstances and environments associated with either public drinking or gambling are likely to increase a person’s risk for engaging in the other activity. Further, more intense treatment may be required for comorbid patients because they are likely to have more functional impairment and a poorer prognosis than are those with either condition alone (Bukstein et al. 1989).

## Future Research

Prospective studies of the temporal association of AUD and PG are virtually absent from the literature but would be best suited to the important goal of clarifying the natural history and temporal relationship of these disorders. It would be important for such studies to capture people early in the window of risk for both disorders (e.g., early adolescence; see above). Also, because one’s drinking or gambling status can change rapidly, the prospective assessments necessary to document important interactions between these conditions should be appropriately frequent (e.g., at 6-month intervals). Although prospective studies like these are difficult and expensive to conduct, reliable information concerning key interactions between gambling and drinking behavior may not be otherwise obtainable.

Another question that has not yet been adequately addressed through research is whether different subtypes of PG and AUD are more likely than others to manifest as a comorbid disorder. For example, some evidence indicates that strong subjective urges of the type linked to specific brain regions (as described above) are an important dynamic in the motivation to gamble among some, but not all, people with PG (Kim et al. 2001). It may be that this subgroup, more than others with PG, are likely to develop comorbid substance use disorders. That is, some disorder subtypes may be associated with neurobiological processes that overlap for both PG and AUD, whereas other subtypes of these disorders may have a different etiology that is unrelated to the comorbid condition. Research findings from genetic and brain imaging studies will further help identify key subgroup variables and serve as a methodology for identifying common biological substrates associated with both PG and AUD.

In addition to studies aimed at unraveling the shared and unique etiologies of these conditions, research must also seek to develop and test treatment and prevention strategies. For example, it is currently unknown whether parallel, serial, or integrated treatment approaches would best serve the comorbid population. Given that the bulk of systematic research into the psychosocial and pharmacological treatment of PG has been ongoing for less than 10 years, however, it is not surprising that a lot of work still needs to be done in this area (Kim and Grant 2001).

Yet another area of potential inquiry is that of commonalities in personality dimensions between people with PG and those with AUD. PG and AUD share features of impulsivity and behavioral excess. Whether these disorders share some common personality traits or a categorical personality disorder is currently unknown. Future research is needed to assess the personality dimensions of those who engage in these addictive behaviors and determine whether shared personality traits may have possible treatment implications.

Finally, additional experimental studies may be needed to map more precisely the impact of drinking on gambling behavior and vice versa (e.g., see Stewart et al. 2000). As reviewed, only a very small number of such studies have been conducted and these have produced mixed findings. Clearer trends would be expected to emerge from a larger number of studies. These studies have the capacity to systematically manipulate myriad variables that may influence this relationship (e.g., gender, dose of alcohol, type of gambling decision/behavior, degree of urge driving the gambling behavior, stakes). Although naturalistic study of such factors might not be practical, experimental studies have the potential to produce results that bear directly on processes related to comorbidity.

## Conclusions

PG frequently co-occurs with AUD. The preponderance of the available data suggests that overlapping brain systems may leave people vulnerable to both disorders. However, some researchers have speculated that the causal influences in PG and AUD may be heterogeneous. That is, some common causal influences shared by AUD and some subtypes of PG may promote comorbidity, but the causes and maintaining influences of other subtypes of PG and AUD may not overlap. From a clinical perspective, findings reviewed here highlight how important it is for AUD treatment programs to provide careful screening and treatment planning that is capable of recognizing and reacting to the presence of comorbid PG. From a scientific standpoint, understanding comorbidity will necessarily refine views on the psychopathology and taxonomy of the two conditions. Hopefully, reviews such as this will encourage both gambling and alcohol use researchers and clinicians, where possible, to expand their scientific and clinical enterprises to explicitly include comorbid cases.
